# Atrial Fibrillation Initiated by Early Afterdepolarization-Mediated Triggered Activity during Acute Oxidative Stress: Efficacy of Late Sodium Current Blockade

**DOI:** 10.16966/2379-769X.146

**Published:** 2018-04-25

**Authors:** Arash Pezhouman, Hong Cao, Michael C Fishbein, Luiz Belardinelli, James N Weiss, Hrayr S Karagueuzian

**Affiliations:** 1 Translational Arrhythmia Section, UCLA Cardiovascular Research Laboratory, USA; 2 Departments of Medicine (Cardiology), David Geffen School of Medicine at UCLA, Los Angeles, California, USA; 3 Department of Cardiology, Renmin Hospital of Wuhan University, Wuhan, PRC; 4 Department of Pathology, David Geffen School of Medicine at UCLA, USA; 5 InCarda Therapeutics, Brisbane, California, USA

**Keywords:** Atrial fibrillation, CAMKII, Fibrosis, Early afterdepolarizations, Optical activation map, Hydrogen Peroxide, GS-967, Late inward sodium current

## Abstract

**Background:**

The mechanism of Atrial Fibrillation (AF) that emerges spontaneously during acute oxidative stress is poorly defined and its drug therapy remains suboptimal. We hypothesized that oxidative activation of Ca-calmodulin dependent protein kinase (CaMKII) promotes Early Afterdepolarization-(EAD)-mediated triggered AF in aged fibrotic atria that is sensitive to late Na current (I_Na-L_) blockade.

**Method and Results:**

High-resolution voltage optical mapping of the Left and Right Atrial (LA & RA) epicardial surfaces along with microelectrode recordings were performed in isolated-perfused male Fisher 344 rat hearts in Langendorff setting. Aged atria (23–24 months) manifested 10-fold increase in atrial tissue fibrosis compared to young/adult (2–4 months) atria (P<0001. Spontaneous AF arose in 39 out of 41 of the aged atria but in 0 out of 12 young/adult hearts (P<001) during arterial perfusion of with 0.1 mm of hydrogen peroxide (H_2_O_2_). Optical Action Potential (AP) activation maps showed that the AF was initiated by a focal mechanism in the LA suggestive of EAD-mediated triggered activity. Cellular AP recordings with glass microelectrodes from the LA epicardial sites showing focal activity confirmed optical AP recordings that the spontaneous AF was initiated by late phase 3 EAD-mediated triggered activity. Inhibition of CaMKII activity with KN-93 (1 μM) (N=6) or its downstream target, the enhanced I_Na-L_ with GS-967 (1 μM), a specific blocker of I_Na-L_ (N=6), potently suppressed the AF and prevented its initiation when perfused 15 min prior to H_2_O_2_ (n=6).

**Conclusions:**

Increased atrial tissue fibrosis combined with acute oxidative activation of CaMK II Initiate AF by EAD-mediated triggered activity. Specific block of the I_Na-L_ with GS-967 effectively suppresses the AF. Drug therapy of oxidative AF in humans with traditional antiarrhythmic drugs remains suboptimal; suppressing I_Na-L_ offers a potential new strategy for effective suppression of oxidative human AF that remains suboptimal.

## Introduction

Oxidative stress has been shown to increase the susceptibility of the heart to ventricular and atrial fibrillation (VF and AF respectively) in animal models that manifest increased cardiac fibrosis [1,2]. Increased systemic and atrial myocardial oxidative stress is often observed in post-operative patients with new onset (acute) paroxysmal AF (POAF) [3,4] with an incidence of up to 50% [5]. Diverse etiological factors such as fibrosis [6,7] and increased Ca^2+^/calmodulin-dependent protein kinase II (CaMKII) activity [8,9] among others, are thought to play a key role in the initiation of oxidative AF [4,10]. While the causative and signaling factors of oxidative AF are reasonably well identified, the mechanism of spontaneous initiation (i.e., not induced by electrical stimulation) of acute oxidative AF in experimental and human studies, remains undefined [10]. The lack of mechanistic cellular insight into the genesis of oxidative AF hampered the development of effective pharmacological therapy to suppress and/or prevent the oxidative AF [11]. For example, beta-blockers [12] and Amiodarone, [13] considered first line preventive drugs against POAF, are only partially effective. Interestingly, both human and animal studies have shown increased atrial CaMKII activity as the molecular signal that couples oxidative stress with AF [10]. Activated CaMKII phosphorylates cardiac Na and Ca channels enhancing the highly arrhythmogenic late Na (I_Na-L_) [14] and the late L-type Ca (I_Ca-L_) [15] currents. Isolated myocytes and dynamic-clamp experiments have shown that an isolated increase of either of these two late inward currents promotes cellular early and delayed afterdepolarizations (EADs and DADs respectively) causing rapid triggered activity [16,17]. Indeed inhibition of the I_Na-L_ with the highly potent (IC_50_=0.143 μM) and selective inhibitor GS-967 [18] suppresses EADs in isolated atrial myocytes [18,19]. The purpose of this study is to test the following two hypotheses; 1) oxidative stress-mediated activation of CaMKII signaling with hydrogen peroxide (H_2_O_2_) in structurally remodeled atria characterized with increased atrial tissue fibrosis promotes AF by the mechanism of EAD and DAD-mediated triggered activity; and 2) selective inhibition of the enzymatic activity of CaMKII or its distal target, the I_Na-L_ with the specific I_Na-L_ blocker, GS-967, [18] suppresses the oxidative AF.

## Methods

Our study protocol conforms to the Guide for the Care and Use of Laboratory Animals and is approved by our Institutional Animal Use and Care Committee.

### Langendorff preparation

#### Optical mapping and microelectrode recording

We used male Fisher 344 rats. The isolated hearts were arterially perfused with oxygenated normal Tyrode’s solution. The hearts were stained with the voltage-sensitive fluorescent dye, RH-237 for optical activation mapping of the epicardial surfaces of both the LA and the RA appendages. We use a CMOS camera (MiCAM Ultima, Brain Vision, Tokyo, Japan) at 1 ms/frame and 100 × 100 pixels with a spatial resolution of 0.35 × 0.35 mm^2^/pixel. Cytochalasin D (5 μmol/l) was added to the perfusate to eliminate motion artifacts during optical recordings [20,21]. Sites of frequent atrial epicardial focal activity arising at the onset of AF detected with the optical mapping were subsequently probed with single cell glass microelectrode recordings to determine the cellular mechanism of the underlying focal activity.

#### Histological analyses of atrial tissue fibrosis

The hearts of the young and the old rats were fixed in 4% buffered formalin for 1 hour and then placed in 70% alcohol. Longitudinal and transverse sections (5 μm thickness) were made in the LA appendage and the free wall and stained with trichrome that stain collagen fibers blue. Percent LA fibrosis was determined as we previously described [7,20].

#### Statistical analyses

Significant differences in the incidence of AF (dichotomous comparisons) were determined using Fisher’s exact test. The APDs were determined using repeated-measures ANOVA. We consider *P* value of <0.05 as significant. Data are presented as means ± SD.

## Results

### Increased fibrosis in atria from aged *versus* young/adult atria

**Figure 1:**
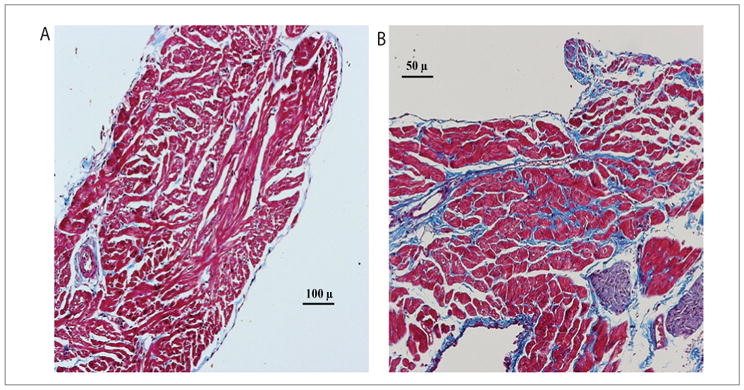
Histological sections of trichrome staining of LA epicardial appendages in a young/adult (A) *versus* an old (B) rat heart. Note the increased interstitial fibrosis (blue stain) in the old rat myocardium, causing separation of myocardial bundles (stained red).

A significant and highly heterogeneous increase in the LA and RA interstitial and replacement tissue fibrosis was observed in atria of aged (22–24 months) as compared to young/adult (2–4 months) rats (12.7 ± 10.6 *vs.* 1.4 ± 0.6, *P*<0.001) (Figure 1). These findings are consistent with our previous findings in aged rats and rabbits atria [1,7,20,22] as well as in aged human atria [23].

### Spontaneous AF initiation in aged rat hearts during acute oxidative stress caused by arterial perfusion of H_2_O_2_: The role of CaMKII signaling

**Figure 2:**
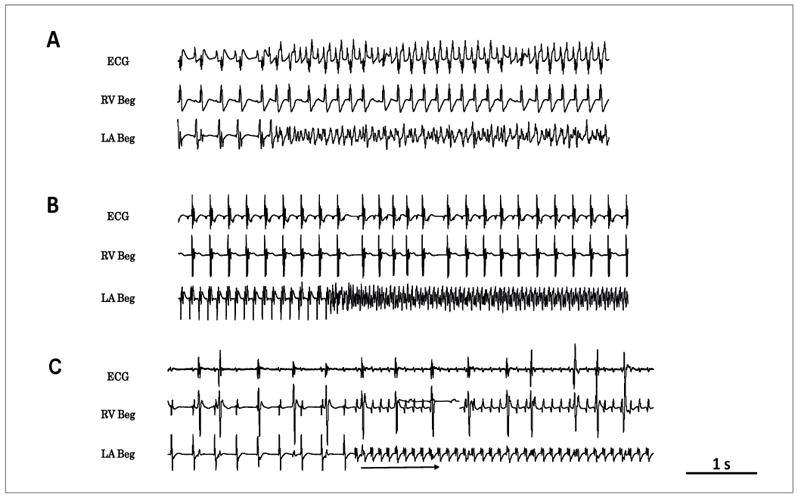
Patterns of spontaneous initiation of AF in isolated-perfused aged hearts exposed to H_2_O_2_ (hydrogen peroxide) (0.1 mM) in three different aged hearts. Panel A shows sudden onset of AF arising during sinus rhythm. In panel B, AF starts after a prior transient period of AT at a CL of 120 ms and panel C shows the sudden emergence of AT at a cycle length of 116 ms that last more than 30 sec before degenerating to AF.

**Figure 3:**
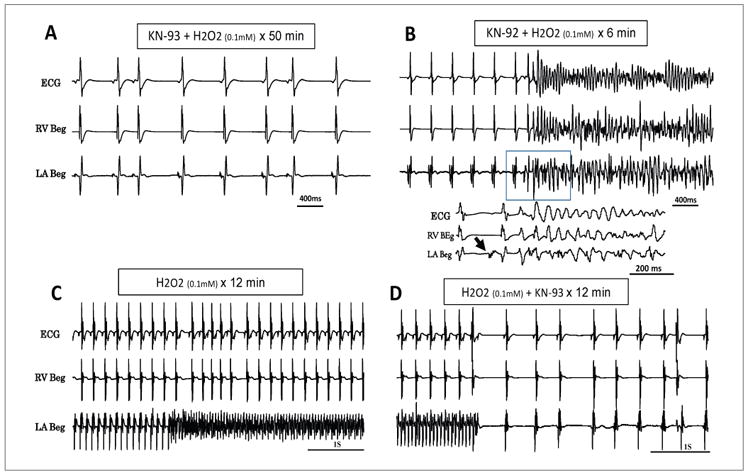
Prevention and suppression of acute oxidative AF with the CaMKII inhibitor KN-93 (1 μM). Pretreatment with KN-93 prevents emergence of H_2_O_2_-induced AF over a 50 min of observation (panel A). However, subsequent replacement of KN-93 with its inactive form, KN-92 (1 μM) causes AF to emerge within 6 minutes of the switch (panel B). Note below the panel B a faster sweep recording showing the onset of AF (arrow) followed immediately by VF. Panel C shows another aged heart in which the AF was initiated with H_2_O_2_ (0.1 mM) and was effectively suppressed by the addition of KN-93(1 μM) in the continuous presence of H_2_O_2_

Because oxidative stress with H_2_O_2_ also promotes VF in aged hearts [1] we determined the mechanism(s) of spontaneous AF initiation of AF only when the AF preceded the VF or when the AF was not associated with VF as shown in figure 2. Under these conditions the AF emerged after a mean of 12 ± 7 min exposure to H_2_O_2_ and was often preceded by a transient period of monomorphic atrial tachycardia or flutter (AT/AFl) at a mean cycle length (CL) of 80 ± 46 ms (Figure 2). The transient AT then suddenly degenerates to a very rapid (CL of 45 ± 12 ms) and irregular atrial electrogram activity indicating the transition of AT/AFl to AF (Figure 2). We then determined the role of atrial CaMKII activity on H_2_O_2_-mediated AF. For this purpose, we first perfused the hearts of aged rats (N=6) with the specific CaMKII inhibitor KN-93 (1 μM) for 15 min and then added 0.1 mM H_2_O_2_ to the perfusate in the continuous presence of KN-93. No AF emerged for one hour of observation in any of the six aged hearts studied (Figure 3). At this point, we replaced the KN-93 with its inactive form KN-92 (1 μM) in the continuous presence of H_2_O_2_ to determine if off target effects of KN-92 played any role in the AF suppression. The switch caused the emergence of AT/AF in all six hearts after 8 ± 4 mins (Figure 3). In six additional hearts, KN-93 (1 μM) was added after the onset of H_2_O_2_-induced AF. KN-93 terminated AF in all six hearts after a mean exposure time of 14 ± 7 mins (Figure 3). These findings indicate that activation of atrial CaMKII signaling pathway plays a key role in the initiation H_2_O_2_-mediated AF in aged rat hearts characterized by increased atrial tissue fibrosis.

### H_2_O_2_ fails to induce atrial arrhythmias in hearts of young/adult rats

In contrast to the susceptibility of aged atria to oxidative AF, arterial perfusion of 0.1 mM H_2_O_2_ in young/adult hearts for up to 90 mins failed to induce AF in all 6 hearts studied. Furthermore, elevation of the H_2_O_2_ concentration for up to 2 mM (N=5) in the young/adult hearts still failed to promote EADs and/or atrial ectopic activity consistent with our previous findings [1,7,22] and by Lin CS et al. [24]. The discrepancy between the ease of oxidative EAD formation at the single myocyte level isolated from young/adult atria [18,25] and the resistance to EAD formation at the atrial tissue level in young/adult hearts, suggests that atrial tissue factor(s) must have been responsible in the aged tissue to readily promote EAD and EAD-mediated triggered activity. These findings suggest that increased atrial tissue fibrosis observed in the aged atria may indeed be a key factor in facilitating the formation EADs and EAD-mediated AF.

### Optical activation map and single cell microelectrode recordings during the onset of spontaneous AF

**Figure 4:**
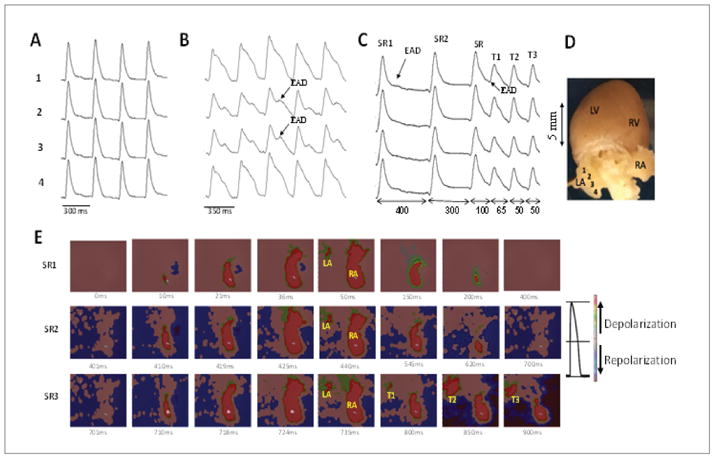
Optical APs (OAPs) and voltage snapshots of epicardial LA and RA activation during sinus rhythm and at the onset of oxidative AF in an aged rat heart. Panel A shows four rows of OAPs recorded from the LA epicardial surface from sites shown in panel D during perfusion with normal Tyrode’s. Panel B shows the influence of 8 min of perfusion with H_2_O_2_ (0.1 mM) on these OAPs with the emergence of subthreshold EADs. Panel C shows the last three sinus beats just before the onset of EAD-mediated triggered activity (T1–T3). Note that the EAD amplitude during the sinus beat#1 and #2 manifest subthreshold EAD (arrows). However, the 3^rd^ sinus beat initiates triggered activity (T1–T3). Double-headed arrows indicate the CL of the specified intervals. Panel E shows the snap shots of the six beats SR1, SR2, SR3, T1, T2, and T3, with the origination of the sinus beats from the RA and triggered beats arising from the LA. Adjoining the maps is a color bar showing depolarization and repolarization respectively.

**Figure 5:**
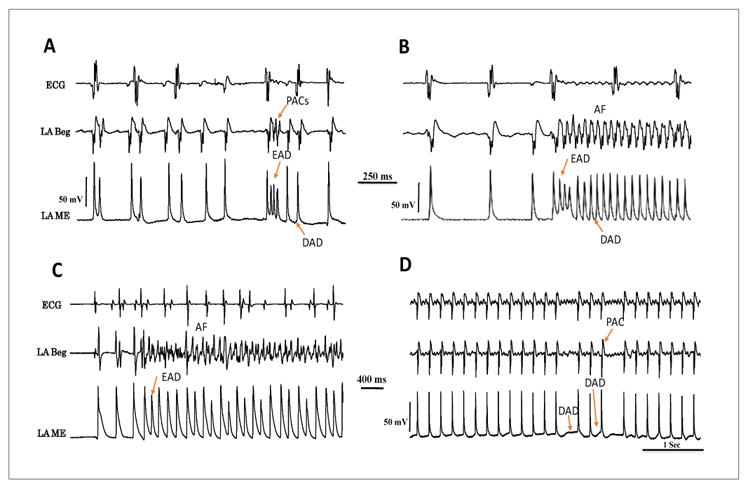
Glass microelectrode recordings of LA epicardial APs showing Premature Atrial Complexes (PAC) in panel A; atrial tachycardia (panel B) and AF (panel C) initiated with H_2_O_2_ (0.1 mM). Notice the onset of these arrhythmias by EAD-mediated triggered beats. While DADs also appear after a prior run of EAD-mediated triggered activity DAD-mediated causing triggered (Panels A and B), DADs cause only single triggered beats (PAC) as shown in panel D.

We successfully captured atrial epicardial activation pattern at the onset of spontaneous oxidative AF in three hearts. Optical action potentials (Figure 4A) manifest a progressive emergence of late phase EAD-like activity on the LA epicardial appendage (Figure 4B). These sub thresholds EADs subsequently triggered rapid repetitive activity causing rapid focal activity on the LA as observed on the optical activation map (Figures 4B and 4C). The focal activity than propagated as a target wave over the LA epicardial surface eventually degenerating to disorganized wavefront activity that signaled the sudden onset of AF (Figures 4B and 4C). Figure 4D shows the isolated rat heart with locations of the right and left atrial appendages (RA and LA respectively as well as the right and the left ventricles (RV and LV). Panel D shows optical action potential map with and adjacent color bar showing depolarization and repolarization respectively. In additional five aged hearts continuous single cell glass microelectrode recordings from the LA epicardial appendages showing focal activity at the onset of AF initiation determined with optical activation maps, confirmed that the focal activity was indeed initiated by cellular late phase 3 EAD-mediated triggered activity as shown in figures 5A–C. After the onset of EAD-mediated AF continuous microelectrode recordings showed the emergence of DADs which then in turn initiated DAD-mediated triggered activity contributing to the maintenance of AF as shown in figure 5. Synergistic interaction between the EADs and the DADs (EAD promoting DAD and DAD promoting EADs) was convincingly demonstrated in ventricular myocytes and simulated ventricular myocytes [26]. The present result extends this phenomenon to atrial tissue as well. Interestingly the emergence of DADs in the atrial tissue (Figure 5) caused only single premature atrial complexes (PAC) but failed to initiate triggered activity and AF. DAD-mediated triggered activity required priming by a prior EAD-mediated triggered activity, a phenomenon help maintain the AF.

### Suppressive and preventive effects of GS967 against oxidative AF in aged atria

**Figure 6:**
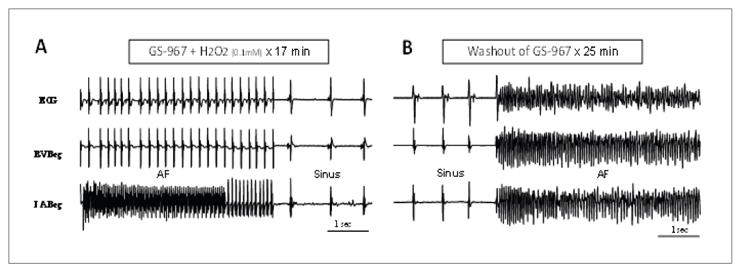
Suppression of H_2_O_2_-mediated AF with 1 μM GS-967 Notice an initial transition from AF to rapid AT before converting to sinus rhythm (panel A). Panels B demonstrates that the anti-AF effect of the GS-967 is reversible as AF re-emerges upon the washout of the drug. Also notice the simultaneous emergence of oxidative VF upon the washout of the GS-967 (panel B).

The addition of GS-967 in the perfusate 15 min prior to exposure to H_2_O_2_ prevented AF initiation in 6 out of 6 hearts studied for up to one hour of observation. However, upon washout of GS-967, AT/AF emerged in all six hearts after a mean of 16 ± 4 mins. GS-967 also suppressed the AF after it was initiated with H_2_O_2_ in six out six aged hearts after a mean perfusion time of 14 ± 6 mins (Figure 6C). The effect if GS-967 was reversible as AF reemerged after a mean of 21 ± 7 mins of GS-967 washout (Figure 6D).

### Effects of H_2_O_2_ and GS-967 on atrial action potential duration (APD) in young/adult and aged rat atria

There were no significant differences (P>0.3) in the APD to 90 percent repolarization (APD_90_) between the young/adult and aged left atrial appendages at baseline (54 ± 6 ms *vs*. 55 ± 10 ms) (Table 1). Arterial perfusion with 0.1 mM H_2_O_2_ significantly prolonged (P<0.01) atrial APD_90_ in both age groups, 100 ± 8 ms *vs*. 105 ± 12 respectively. (N=6 in each group, PCL=400 ms) (Table 1). The addition of GS-967 normalized the APD_90_ in both age groups exposed to H_2_O_2_ (56 ± 8 ms and 56 ± 10 ms) in the young/adult and the aged group respectively) (Table 1).

## Discussion

### Novel findings

The major findings of this study are as follows: 1) acute oxidative stress with H_2_O_2_ in isolated-perfused aged atria readily promotes spontaneous AF *via* the activation of CaMKII signaling pathway; 2) oxidative stress with similar or even 10 time higher levels of H_2_O_2_ fail to promote AF in the young/adult atria; 3) left atrial epicardial transmembrane APD_90_ was not significantly different between the two age groups both before and after arterial perfusion of H_2_O_2_ or after the combined perfusion of H_2_O_2_+GS-967; 4) the mechanism of oxidative AF initiation is caused by cellular EAD-mediated triggered activity; 5) oxidative AF is prevented and suppressed by either direct enzymatic inhibition of CaMKII activity (proximal target) or by downstream inhibition of CaMKII-mediated enhanced I_Na-L_ by GS-967. These results provide a novel cellular insight into the mechanism of spontaneous initiation (not electrically-induced) of oxidative AF that was not reported previously.

### Oxidative AF: substrate, mechanisms and signaling

Our results highlight that the combined presence of both atrial tissue fibrosis and acute oxidative stress are necessary for the initiation of spontaneous AF. Neither fibrosis alone (aged atria perfused with normal Tyrode’s solution) nor acute oxidative stress alone (peroxide perfusion in non-fibrotic young/adult atria) is sufficient to promote AF. This suggests a synergistic interaction between oxidative stress and increased atrial tissue fibrosis in the promotion of spontaneous AF [27]. Indeed young/adult isolated atrial myocytes readily manifest EADs and TA at the isolated atrial myocyte level [18,28] however, not at tissue level as shown in this study. Simulation studies [29] have convincingly demonstrated the importance of electrotonic repolarizing “sink” effect caused by well-coupled cells that prevents initiation of EAD-mediated arrhythmias in normal well-coupled atrial tissue. Increased interstitial fibrosis diminishes repolarizing “sink” effect by decreased cellular coupling [30] allowing the EADs to emerge and propagate in the tissue [29]. Indeed in the present study we found no difference in the APD between the two age groups, suggesting no major role played by electrical remodeling in the enhanced sensitivity to AF in the aged atria. This finding further strengthens the major role played by increased atrial tissue fibrosis as a substrate and trigger in the promotion of oxidative EAD-mediated AF.

The potent suppressive effect of GS-967 against oxidative AF provides a novel class of antiarrhythmic drug action not considered in previous drug action classification [11]. Interestingly, recent studies demonstrated, consistent with the results of the present study, that increased I_Na-L_ plays a key role in the induction of rapid pacing-induced AF in mice and its suppression by GS-967 [31]. The importance of I_Na-L_ blockade in the suppression of AF with the novel specific I_Na-L_ blocker, eleclazine was also demonstrated in larger animals (pigs) [32] indicating the potential antiarrhythmic efficacy of this new class of drug action in humans [11].

### Clinical implications

The demonstration of cellular EAD-mediated new onset AF during acute oxidative stress provides an important clinical translational value. It is quite difficult, if not impossible, to obtain reliable recordings of cellular events in intact human hearts at the onset of AF [33]. Human clinical studies demonstrated the occurrence of new onset acute oxidative AF by showing that inhalation of pro-oxidant particulate matters promotes acute AF in patients with cardiac disease [34] and that such effects can be minimized by antioxidant therapy [35]. Genomic analyses showed that genes that were uniquely up regulated in patients with no POAF were mostly supported by reduction reactions suggesting that the overall redox balance favors anti-oxidant state in these patients resistant to POAF [3]. Indeed, mice lacking critical oxidation sites in CaMKII or mice over expressing methionine sulfoxide reductase A, an enzyme that reduces oxidized CaMKII, are resistant to oxidative AF evaluated by programmed electrical stimulation [10]. Recent clinical studies showed that POAF originated from the LA and not from the pulmonary veins (PVs) [36,37] as in the present study. Furthermore it was shown in post-surgical patients manifesting increased atrial tissue fibrosis (confirmed by biopsies) facilitated the emergence LA foci (“sources”) leading to AF [6,25]. That indeed the EAD-mediated triggered activity is the final path to AF initiation is emphasized by suppressing the AF by the selective block of the I_Na-L_ with GS-967 a powerful and selective inhibitor of EADs. Finally, the additional activation of the CaMKII signaling [38] secondary to the elevation of cytosolic Ca^2+^ during the AF further promotes EADs by increasing enhanced I_Na-L_ in a positive feedback loop [38].

### Limitations

Although reduction of electrical load caused by fibrosis provides a plausible explanation for the increased susceptibility of aged hearts to oxidative EAD formation and EAD-mediated AT/AF, we cannot exclude the possibility that other aspects of aging-related remodeling (e.g., altered Ca_i_^2+^ cycling) may also make important contributions. Indeed, human atrial myocytes isolated from patients with AF show increased CaMKII-dependent phosphorylation of RyR2 leading to increased SR Ca^2+^ leak and elevated cytosolic Ca^2+^ levels [39]. These altered subcellular calcium dynamics promotes EADs and DADs formation [26] in a positive feedback loop [38] promoting AF. Finally, the validity of H_2_O_2_ as clinically relevant oxidative stress may be questioned. However as stressed previously [40] levels 100–150 μM concentrations of H_2_O_2_ have been suggested to be clinically relevant to mimic reversible pro-oxidant pathological states [41]. Indeed H_2_O_2_ has been used successfully to assess the influence of oxidative stress in AF in diverse animal models [23,25,42,43].

## Figures and Tables

**Table 1:** Effects of GS-967 on APD_90_ during baseline and after H_2_O_2_

	Young	Aged	*p*-value
	APD 90%	APD 90%	
Baseline	55 ± 10 ms	54 ± 6	0.67
GS-967	28 ± 8 ms*	31 ± 4*	0.1
H_2_O_2_	105 ± 12 ms×	100 ± 8 ms×	0.08
H_2_O_2_+GS-967	56 ± 10 ms*	58 ± 8*	0.38

* p<0.05

× p<0.01

All values are determined during Pacing Cycle Length (PCL) of 400 ms. The symbols * and ^×^ indicate the level of significance. H_2_O_2_ and GS-967 concentrations are 0.1 MM and 1 μM respectively. All values are mean ± SD. N=6 in each group.

## Abbreviations

AF: Atrial Fibrillation
APD: Action Potential Duration
AT/AFl: Atrial Tachycardia and Atrial Flutter Respectively
Beg: Bipolar Electrogram
CaMKII: Ca^2+^/Calmodulin-Dependent Protein Kinase II
CL: Cycle Length
EAD/DAD: Early and Delayed Afterdepolarization Respectively
PCL: Pacing Cycle Length
I_Na-L_: Late Na Current
POAF: Post-Operative Atrial Fibrillation
PAC: Premature Atrial Complex
RA and LA: Right And Left Atria Respectively
VT/VF: Ventricular Tachycardia and Fibrillation Respectively

## References

[1] Morita N, Sovari AA, Xie Y, Fishbein MC, Mandel WJ (2009). Increased Susceptibility of Aged Hearts to Ventricular Fibrillation During Oxidative Stress. Am J Physiol Heart Circ Physiol.

[2] Sakabe M, Fujiki A, Sakamoto T, Nakatani Y, Mizumaki K (2012). Xanthine oxidase inhibition prevents atrial fibrillation in a canine model of atrial pacing-induced left ventricular dysfunction. J Cardiovasc Electrophysiol.

[3] Ramlawi B, Otu H, Mieno S, Boodhwani M, Sodha NR (2007). Oxidative stress and atrial fibrillation after cardiac surgery: a case-control study. Ann Thorac Surg.

[4] Zakkar M, Ascione R, James AF, Angelini GD, Suleiman MS (2015). Inflammation, oxidative stress and postoperative atrial fibrillation in cardiac surgery. Pharmacol Ther.

[5] Xiong F, Yin Y, Dube B, Page P, Vinet A (2014). Electrophysiological changes preceding the onset of atrial fibrillation after coronary bypass grafting surgery. PLoS One.

[6] Swartz MF, Fink GW, Lutz CJ, Taffet SM, Berenfeld O (2009). Left *versus* right atrial difference in dominant frequency, K(+) channel transcripts, and fibrosis in patients developing atrial fibrillation after cardiac surgery. Heart Rhythm.

[7] Hayashi H, Wang C, Miyauchi Y, Omichi C, Pak HN (2002). Aging-related increase to inducible atrial fibrillation in the rat model. J Cardiovasc Electrophysiol.

[8] Guo X, Yuan S, Liu Z, Fang Q (2014). Oxidation- and CaMKII-mediated sarcoplasmic reticulum Ca(2+) leak triggers atrial fibrillation in aging. J Cardiovasc Electrophysiol.

[9] Anderson ME (2015). Oxidant stress promotes disease by activating CaMKII. J Mol Cell Cardiol.

[10] Purohit A, Rokita AG, Guan X, Chen B, Koval OM (2013). Oxidized Ca (2+)/Calmodulin-Dependent Protein Kinase II Triggers Atrial Fibrillation. Circulation.

[11] Karagueuzian HS, Pezhouman A, Angelini M, Olcese R (2017). Enhanced Late Na and Ca Currents as Effective Antiarrhythmic Drug Targets. Front Pharmacol.

[12] Connolly SJ, Cybulsky I, Lamy A, Roberts RS, O’Brien B (2003). Double-blind, placebo-controlled, randomized trial of prophylactic metoprolol for reduction of hospital length of stay after heart surgery: The beta-Blocker Length of Stay (BLOS) study. Am Heart J.

[13] Mitchell LB, Exner DV, Wyse DG, Connolly CJ, Prystai GD (2005). Prophylactic Oral Amiodarone for the Prevention of Arrhythmias that Begin Early After Revascularization, Valve Replacement, or Repair: PAPABEAR: a randomized controlled trial. JAMA.

[14] Wagner S, Dybkova N, Rasenack EC, Jacobshagen C, Fabritz L (2006). Ca^2^+/calmodulin-dependent protein kinase II regulates cardiac Na^+^ channels. J Clin Invest.

[15] Xie LH, Chen F, Karagueuzian HS, Weiss JN (2009). Oxidative Stress-Induced Afterdepolarizations and Calmodulin Kinase II Signaling. Circ Res.

[16] Madhvani RV, Angelini M, Xie Y, Pantazis A, Suriany S (2015). Targeting the late component of the cardiac L-type Ca2+ current to suppress early afterdepolarizations. J Gen Physiol.

[17] Morita N, Lee JH, Xie Y, Sovari A, Qu Z (2011). Suppression of re-entrant and multifocal ventricular fibrillation by the late sodium current blocker ranolazine. J Am Coll Cardiol.

[18] Belardinelli L, Giles WR, Rajamani S, Karagueuzian HS, Shryock JC (2015). Cardiac late Na current: Proarrhythmic effects, roles in long QT syndromes, and pathologic relationship to CaMKII and oxidative stress. Heart Rhythm.

[19] Sicouri S, Glass A, Bellardinelli L, Antzelevitch C (2008). Antiarrhythmic effects of ranolazine in canine pulmonary vein sleeve preparations. Heart Rhythm.

[20] Ono N, Hayashi H, Kawase A, Lin SF, Li H (2007). Spontaneous atrial fibrillation initiated by triggered activity near the pulmonary veins in aged rats subjected to glycolytic inhibition. Am J Physiol Heart Circ Physiol.

[21] Bapat A, Nguyen TP, Lee JH, Sovari AA, Fishbein MC (2012). Enhanced Sensitivity of Aged Fibrotic Hearts to Angiotensin II- and Hypokalemia-Induced Early afterdepolarizations-Mediated Ventricular Arrhythmias. Am J Physiol Heart Circ Physiol.

[22] Sato D, Xie LH, Sovari AA, Tran DX, Morita N (2009). Synchronization of chaotic early afterdepolarizations in the genesis of cardiac arrhythmias. Proc Natl Acad Sci U S A.

[23] Lin YK, Chen YA, Lee TI, Chen YC, Chen SA (2017). Aging Modulates the Substrate and Triggers Remodeling in Atrial Fibrillation. Circ J.

[24] Lin CS, Pan CH (2008). Regulatory mechanisms of atrial fibrotic remodeling in atrial fibrillation. Cell Mol Life Sci.

[25] Lin YK, Lin FZ, Chen YC, Cheng CC, Lin CI (2010). Oxidative stress on pulmonary vein and left atrium arrhythmogenesis. Circ J.

[26] Song Z, Ko CY, Nivala M, Weiss JN, Qu Z (2015). Calcium-voltage coupling in the genesis of early and delayed afterdepolarizations in cardiac myocytes. Biophys J.

[27] Karagueuzian HS (2016). Synergism between Enhanced Late Inward Currents and Tissue Fibrosis in the Initiation of Spontaneous Ventricular Tachyarrhythmias. J Heart Health.

[28] Luo A, Ma J, Song Y, Qian C, Wu Y (2014). Larger late sodium current density as well as greater sensitivities to ATX II and ranolazine in rabbit left atrial than left ventricular myocytes. Am J Physiol Heart Circ Physiol.

[29] Xie Y, Sato D, Garfinkel A, Qu Z, Weiss JN (2010). So little source, so much sink: requirements for afterdepolarizations to propagate in tissue. Biophys J.

[30] Spach MS, Dolber PC (1986). Relating extracellular potentials and their derivatives to anisotropic propagation at a microscopic level in human cardiac muscle. Evidence for electrical uncoupling of side-to-side fiber connections with increasing age. Circ Res.

[31] Zhang Y, Wang HM, Wang YZ, Zhang YY, Jin XX (2017). Increment of late sodium currents in the left atrial myocytes and its potential contribution to increased susceptibility of atrial fibrillation in castrated male mice. Heart Rhythm.

[32] Fuller H, Justo F, Nearing BD, Kahlig KM, Rajamani S (2016). Eleclazine, a new selective cardiac late sodium current inhibitor, confers concurrent protection against autonomically induced atrial premature beats, repolarization alternans and heterogeneity, and atrial fibrillation in an intact porcine model. Heart Rhythm.

[33] Brugada P, Wellens HJ (1985). Early afterdepolarizations: role in conduction block, “prolonged repolarization-dependent reexcitation,” and tachyarrhythmias in the human heart. Pacing Clin Electrophysiol.

[34] Link MS, Luttmann-Gibson H, Schwartz J, Mittleman MA, Wessler B (2013). Acute exposure to air pollution triggers atrial fibrillation. J Am Coll Cardiol.

[35] Rodrigo R, Korantzopoulos P, Cereceda M, Asenjo R, Zamorano J (2013). A Randomized Controlled Trial to Prevent Postoperative Atrial Fibrillation by Antioxidant Reinforcement. J Am Coll Cardiol.

[36] Kiaii B, Fox S, Chase L, Fernandes M, Stitt LW (2015). Postoperative atrial fibrillation is not pulmonary vein dependent: Results from a randomized trial. Heart Rhythm.

[37] Seitz J, Bars C, Theodore G, Beurtheret S, Lellouche N (2017). AF Ablation Guided by Spatiotemporal Electrogram Dispersion Without Pulmonary Vein Isolation: A Wholly Patient-Tailored Approach. J Am Coll Cardiol.

[38] Pezhouman A, Singh N, Song Z, Nivala M, Eskandari A (2015). Molecular Basis of Hypokalemia-Induced Ventricular Fibrillation. Circulation.

[39] Neef S, Dybkova N, Sossalla S, Ort KR, Fluschnik N (2010). CaMKII-dependent diastolic SR Ca2+ leak and elevated diastolic Ca2+ levels in right atrial myocardium of patients with atrial fibrillation. Circ Res.

[40] Rhee SG (2006). Cell signaling. H2O2, a necessary evil for cell signaling. Science.

[41] Burgoyne JR, Oka S, Ale-Agha N, Eaton P (2013). Hydrogen peroxide sensing and signaling by protein kinases in the cardiovascular system. Antioxid Redox Signal.

[42] Hanafy DA, Chen YC, Chang SL, Lu YY, Lin YK (2013). Different effects of dronedarone and amiodarone on pulmonary vein electrophysiology, mechanical properties and H2O2-induced arrhythmogenicity. Eur J Pharmacol.

[43] Huang SY, Lu YY, Chen YC, Chen WT, Lin YK (2014). Hydrogen Peroxide Modulates Electrophysiological Characteristics of Left Atrial Myocytes. Acta Cardiol Sin.

